# Pelvic Lymph Node Dissection in Penile Cancer With Inguinal Lymph Node Extranodal Extension: A Multicenter Experience

**DOI:** 10.3389/fsurg.2021.644273

**Published:** 2021-06-15

**Authors:** Zai-Shang Li, Hui Han, Chuang-Zhong Deng, Yong-Hong Li, Chong Wu, Peng Chen, Zhuo-Wei Liu, Zi-Ke Qin, Fang-Jian Zhou

**Affiliations:** ^1^Department of Urology, Shenzhen People's Hospital, The Second Clinic Medical College of Jinan University, Shenzhen, China; ^2^Department of Urology, First Affiliated Hospital of Southern University of Science and Technology, Shenzhen, China; ^3^Minimally Invasive Urology of Shenzhen Research and Development Center of Medical Engineering and Technology, Shenzhen, China; ^4^Department of Urology, Sun Yat-sen University Cancer Center, Guangzhou, China; ^5^State Key Laboratory of Oncology in South China, Guangzhou, China; ^6^Collaborative Innovation Center of Cancer Medicine, Guangzhou, China; ^7^Department of Urology, Affiliated Tumor Hospital of Xinjiang Medical University, Urumqi, China

**Keywords:** cancer management, metastasis, surgery, survival, pelvic lymph node dissection

## Abstract

**Background:** The aim of this study is to determine the necessary extent of penile lymph node dissection (PLND) in penile cancer patients with inguinal lymph node extracapsular extension (ILN-ENE).

**Methods:** Penile cancer patients who underwent PLND in 15 centers from January 2006 to April 2020 were retrospectively analyzed. PLND was performed in patients with ILN-ENE.

**Results:** Sixty-two patients with ILN-ENE were included in the analysis. A total of 51.6% (32/62) of the patients were confirmed to have pelvic lymph node metastasis (PLNM), and 31.3% (10/32) of patients were confirmed to have multiple PLNMs. Of the patients with metastases, 59.4% (19/32) had bilateral inguinal lymph node metastasis (ILNM). According to the anatomical structure, 71.9% (23/32) of the patients had PLNM in the external iliac region, and 56.2% (18/32) had PLNM in the obturator region. Among those with oligo-PLNM, 65.1% (28/43) of the patients had PLNM in the external iliac region and 38.9% (15/43) had PLNM in the obturator region. A significant overall survival difference was observed between patients with the bilateral ILNM and unilateral ILNM (36-month: 21.2 vs. 53.7%, respectively*, P* = *0.023*). Patients with bilateral ILNM had relatively poor metastasis-free survival compared with unilateral ILNM (36-month: 33.0 vs. 13.9%, respectively*, P* = *0.051*).

**Conclusions:** The external iliac and obturator region were the most commonly affected regions in patients with ILN-ENE, and these regions were the only affected regions in patients with oligo-PLNM. Patients with bilateral ILNM had a high risk of PLNM and worse survival.

## Introduction

Pelvic lymph node metastasis (PLNM) in patients with penile cancer results in a poor prognosis (1, 2). The development of lymph node metastasis (LNM) follows the route of anatomical drainage (3). A primary penile tumor first reaches the inguinal lymph nodes (LNs), and then it affects the ipsilateral pelvic LNs (1–4). Pelvic nodal disease does not seem to occur without ipsilateral inguinal LNM (3). Lymphadenectomy (LND) performed in penile cancer patients is therefore critical for locoregional disease control and long-term patient survival (5–7). Based on historical reports, the European Association of Urology (EAU) and the National Comprehensive Cancer Network (NCCN) penile cancer guidelines recommend pelvic lymphadenectomy (PLND) when two or more LNMs or one inguinal node with extranodal extension (ILN-ENE) is observed (1, 2).

The operation time is prolonged by PLND (8). Moreover, the autonomic structure may be altered, and morbidity might be increased (9–11). Although the application of PLND in penile cancer has been reported, the extent of lymphadenectomy is still a subject of substantial debate (5, 6, 12, 13). Detailed maps of LNM in bladder cancer patients with radical cystectomy showed that the scope of LNM increases with stage (11). However, 71% of prostate cancer patients were reported to have LNM located in the obturator fossa and external iliac vessel zone with standard PLND (10).

To our knowledge, the details of the distribution of PLNM in penile cancer patients with ILN-ENE has not been published. The objective of this study was to use multi-center data to determine the optimal anatomic extent of PLND and the outcomes in patients with ILN-ENE.

## Methods

### Study Population

Data from 15 centers were collected for analysis after approval from the medical ethics committee. The data were collected from January 2006 to April 2020. The eligibility criteria were (1) histologically confirmed penile squamous cell carcinoma (PSCC), (2) patients with ILN-ENE, (3) inguinal LND and PLND, and (4) ≥5 removed pelvic LNs. The patients were chosen to be assessed with a surgeon who have performed more than 30 operations. The boundaries of PLND include the distal common iliac, external iliac, obturator, internal iliac artery, and presacral floor. The criteria, boundaries, and technologies associated with our method of inguinal LND have been previously described in detail (12).

PLND was performed in patients with ILN-ENE according to the examination of frozen sections or final postoperative pathology after inguinal lymph node dissection. Prior to January 2009, PLND was performed in cases with clear evidence of solitary pelvic metastasis and with the patient's consent. Since 2009, PLND has been performed in patients with two or more LNMs according to the examination of frozen sections, evidence of ILN-ENE, or pelvic imaging indications. ILNM removed by invasive nodal staging procedures should be included in the final histopathology results. The histopathology results were reported in accordance with the eighth TNM staging system. A fixed or gross nodal mass was defined, and follow-up plans have been previously described in detail (12).

### Statistics

Survival was estimated by the Kaplan–Meier method, and the difference was determined with the log-rank test. Multivariable Cox regression analysis was fitted to test survival predictors. The results are presented as medians and interquartile range (*IQR)* for normally distributed data. Statistical analyses were performed using Statistical Product and Service Solutions software (version 20, SPSS Institute, Chicago, IL, USA).

## Results

### Study Patient Characteristics

In this study, a total of 62 PSCC patients met the inclusion criteria. The median age (*IQR*) was 52.0 (45.0–61.0) years. A total of six (11.1%) and 56 (88.9%) patients had confirmed bilateral ILN-ENE and unilateral ILN-ENE, respectively. Additionally, 54 (87.1%) of the patients underwent bilateral PLND, and eight (12.9%) patients underwent unilateral PLND. The baseline characteristics of the groups are shown in Tables 1, 2.

**Table 1:** Clinical and pathological characteristics of the PSCC patients.

**Variable**	***N* = 62**
Age at surgery, yr, median (IQR)	52.0 (45.0–61.0)
ILNM laterality, No. (%)	
Unilateral	31 (50.0)
Bilateral	31 (50.0)
ENE laterality, No. (%)	
Unilateral	54 (87.1)
Bilateral	8 (12.9)
PLNM, No. (%)	
Yes	32 (51.6)
No	30 (48.4)
Number of PLNM, *n*, median (range)	
1	12 (19.4)
2	8 (12.9)
3	6 (9.7)
≥4	8 (12.9)
PLNM laterality, No. (%)	
Unilateral	27 (43.5)
Bilateral	5 (8.1)
pT, No. (%)	
≤pT1	9 (14.5)
pT2	26 (41.9)
pT3	16 (25.8)
pT4	6 (9.7)
Tx	5 (8.1)
M, No. (%)	
M0	57 (91.9)
M1	5 (8.1)
Grade, No. (%)	
G1	21 (33.8)
G2	32 (51.6)
G3	9 (14.5)
Adjuvant chenmotherapy	
Yes	34 (54.8)
No	28 (45.2)

*ENE, extranodal extension; ILNM, ingunal lymph node metastasis; LN, lymph node; No., number; PLNM, pelvic lymph node metastasis*.

**Table 2:** Baseline characteristics of the PLND.

**Variable**		**Unilateral PLND** **(*n* = 8)**	**Bilateral PLND** **(*n* = 54)**
No. inguinal LNM, No. (%)			
	<4	3 (37.5)	21 (38.9)
	≥4	3 (37.5)	7 (13.0)
	Had Fixed node	2 (25.0)	26 (48.1)
Inguinal LNM laterality, No. (%)			
	Unilateral	4 (50.0)	27 (50.0)
	Bilateral	4 (50.0)	27 (50.0)
ENE laterality, No. (%)			
	Unilateral	8 (100.0)	48 (88.9)
	Bilateral	0	6 (11.1)
No. pelvic LNM, No. (%)	No. Positive	7 (87.5)	25 (46.3)
	0	1 (12.5)	29 (53.7)
	1	2 (25.0)	10 (18.5)
	2	1 (12.5)	5 (9.3)
	3	2 (25.0)	4 (7.4)
	≥4	2 (25.0)	6 (11.1)

*ENE, extranodal extension; LNM, lymph node metastasis; No., number; PLND, pelvic lymph node dissection*.

### Pelvic Lymph Node Distribution

**Figure 1:**
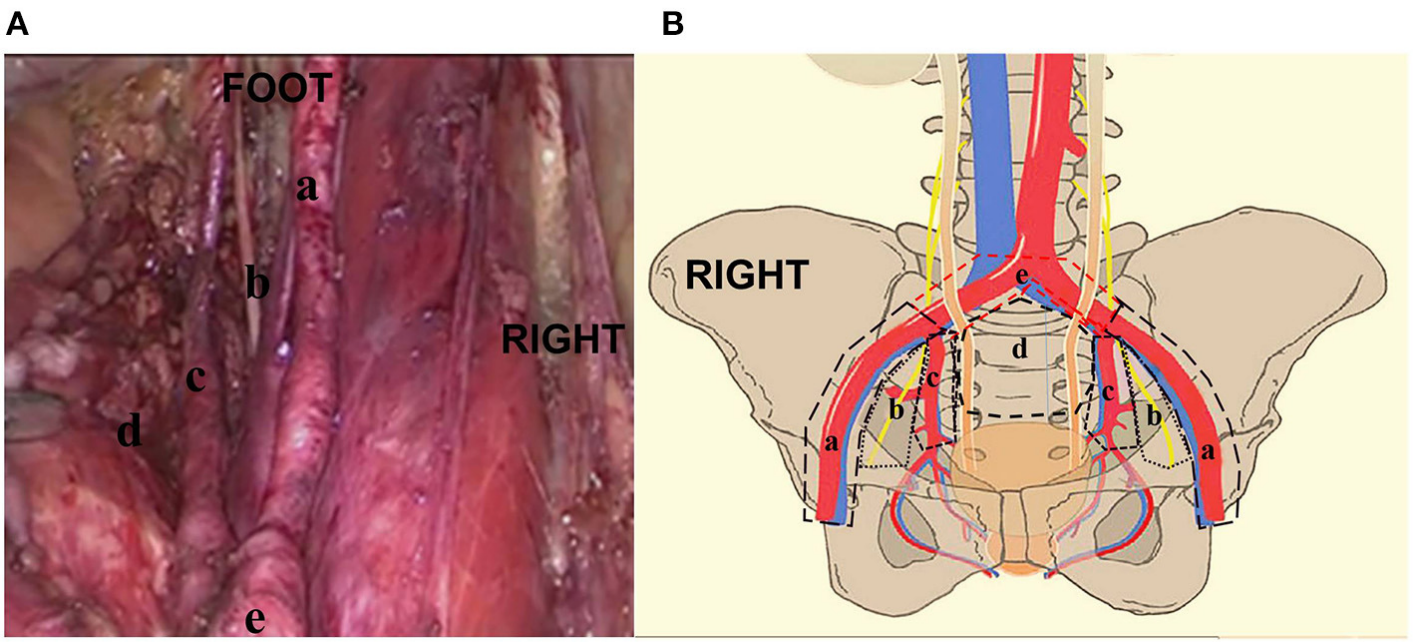
**(A, B)** The distribution of pelvic lymph nodes in the external iliac zone a, obturator zone b, internal iliac zone c, presacral floor zone d, and common iliac zone e. RIGHT: Right side of patient. FOOT: Foot side of patient.

**Figure 2:**
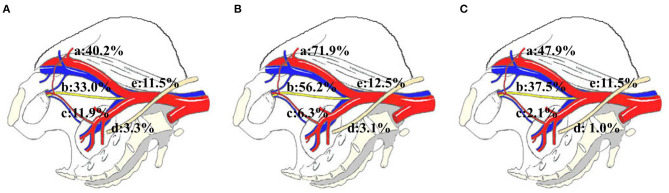
The distribution of pelvic lymph node metastasis in the external iliac zone a, obturator zone b, internal iliac zone c, presacral floor zone d, and common iliac zone e. **(A)** pelvic lymph node. **(B)** PLNM according to the case analysis. **(C)** Pelvic lymph node metastasis according to the distribution analysis.

The distribution and variability of lymph node counts within each zone of dissection are illustrated in Figure 1. The median number of pelvic LNs removed (*IQR*) was 20.0 (14.0–28.0). According to the distribution analysis, 1,277 pelvic LNs were removed, and the external iliac region (40.2%, 513/1,277) and obturator region (33.0%, 422/1,277) were the most commonly harvested regions in patients with ILN-ENE (Figure 2A).

### Pelvic Lymph Node Metastasis Distribution

The median number of PLNMs removed (*IQR*) was 2.0 (1.0–4.0). A total of 51.6% (32/62) of the patients had PLNMs, and 31.3% (10/32) of the patients had multiple PLNMs. Of the patients with metastasis, 59.4% (19/32) had PLNMs with bilateral ILNM. Among the patients with multiple PLNM, 90.0% (9/10) had PLNMs with bilateral ILNM. Only five patients had bilateral PLNM, and no patient had crossover metastatic spread from one inguinal side to the other.

According to the case analysis, 71.9% (23/32) of the cases involved the external iliac region, 56.2% (18/32) involved the obturator region, 12.5% (4/32) involved the common iliac region, 6.3% (2/32) involved the internal iliac region, and 3.1% (1/32) involved the presacral floor region, respectively (Table 3, Figure 2B). Interestingly, in oligo-PLNM, 63.6% (14/22) of the cases involved the external iliac region, and 36.4% (8/22) involved the obturator region (Table 3).

**Table 3:** The distribution of pelvic lymph node metastasis.

**Variable**	**All PLNM patients**		**Oligo-PLNM patients**	
	**Patients** **(*n* = 32)**	**PLNM** **(*n* = 96)**	**Patients** **(*n* = 22)**	**Oligo-PLNM** **(*n* = 43)**
External iliac zone, No. (%)	23 (71.9)	46 (47.9)	14 (63.6)	28 (65.1)
Obturator zone, No. (%)	18 (56.2)	36 (37.5)	8 (36.4)	15 (38.9)
Internal iliac zone, No. (%)	2 (6.3)	2 (2.1)	0	0
Common iliac zone, No. (%)	4 (12.5)	11 (11.5)	0	0
Presacral floor zone, No. (%)	1 (3.2)	1 (1.0)	0	0

*No., number; PLNM, pelvic lymph node metastasis*.

A total number of 96 PLNMs were detected. According to the distribution analysis, 47.9% (46/96) of the cases involved the external iliac region, 37.5% (36/96) involved the obturator region, 11.5% (11/96) involved the common iliac region, 2.1% (2/96) involved the internal iliac region, and 1.0% (1/96) involved the presacral floor region (Table 3, Figure 2C). In oligo-PLNM, 65.1% (28/43) of the cases involved the external iliac region, and 38.9% (15/43) involved the obturator region (Table 3).

### Overall Survival

**Figure 3:**
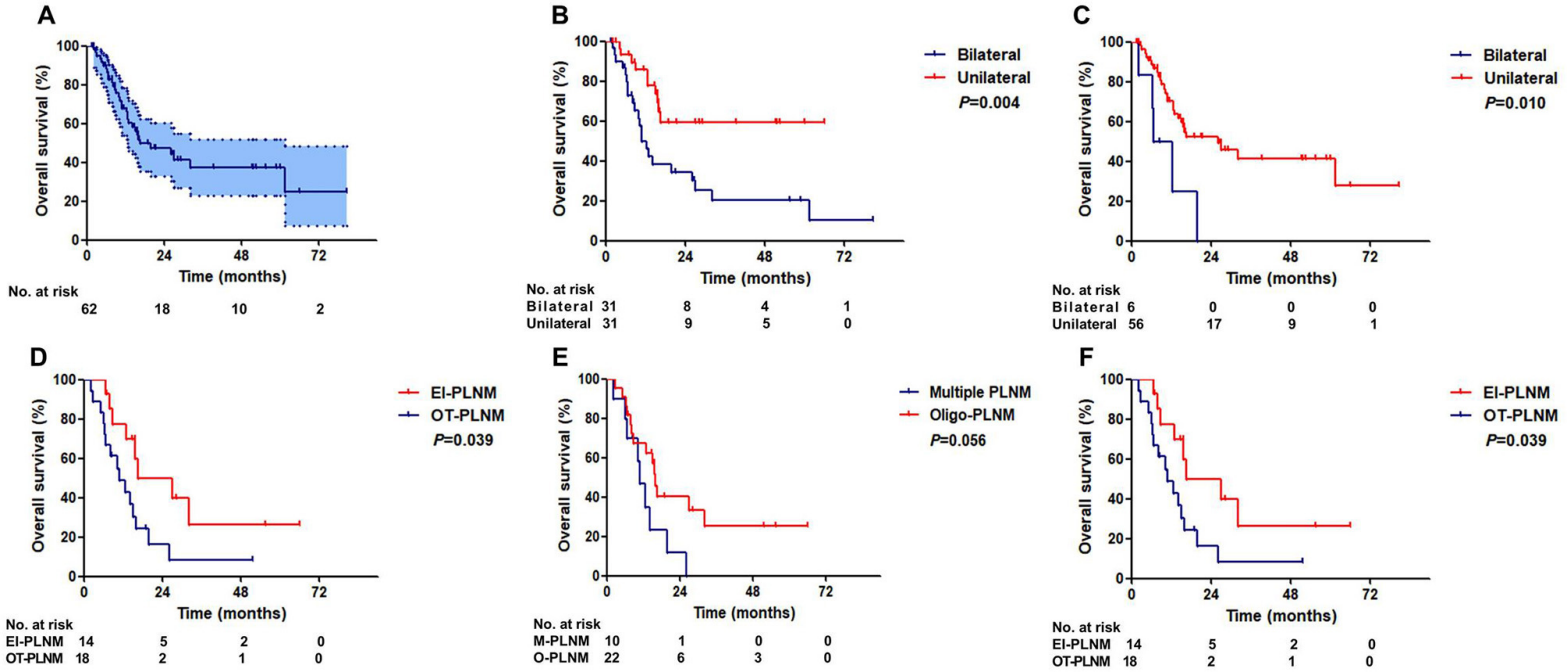
Overall survival of ENE patients. **(A)** All patients. **(B)** Patients with unilateral and bilateral inguinal LNM. **(C)** Patients with unilateral and bilateral ILN-ENE. **(D)** Patients with and without PLNM. **(E)** Patients with multiple PLNM and oligo-PLNM. **(F)** Patients with external iliac region-PLNM and other region-PLNM (EIR-PLNM, external iliac region-PLNM; OR-PLNM, other region PLNM).

The median (*IQR*) follow-up duration was 13.5 (7.5–26.9). The median overall survival (OS) of patients was 16.6 months (*95% CI:3.8–29.3*). The 3-year OS rate was 37.5% (*95% CI: 22.6–52.4%*, Figure 3A).

Patients with unilateral ILNM had a significantly better OS than patients with bilateral ILNM (36-month OS: 20.2 vs. 59.5%, *P* = *0.004*, Figure 3B). Patients with unilateral ILN-ENE exhibited significantly better OS than patients with bilateral ILN-ENE (36-month OS: 41.6% vs. 0, *P* = *0.010*, Figure 3C).

There was a significant difference in survival between the groups of patients with and without PLNM (36-month OS: 67.3 vs. 16.1%, *P* = *0.010*, Figure 3D). Additionally, although the difference was not significant, the patients with multiple PLNMs had worse OS rates than the patients with oligo-PLNM (36-month OS: 25.1% vs. 0, *P* = *0.054*, Figure 3E). However, patients with external iliac region-PLNM had a significantly better OS rate than patients with PLNM in other regions (36-month OS: 26.5 vs. 8.1%, *P* = *0.039*, Figure 3F). Multivariate Cox regression analysis results are shown in Table 4.

**Table 4:** Univariable and multivariate Cox regression of OS and MFS.

**Variable**	**OS**				**MFS**			
	**Univariable**		**Multivariable**		**Univariable**		**Multivariable**	
	***HR***	***P***	***HR***	***P***	***HR***	***P***	***HR***	***P***
ILNM laterality	2.9 (1.3–6.0)	0.01	2.4 (1.1–5.3)	0.03	1.9 (1.0–3.7)	0.04	1.7 (0.9–3.3)	0.13
ENE laterality	3.4 (1.3–9.0)	0.02	1.8 (0.7–5.1)	0.25	2.6 (1.0–6.7)	0.06	1.8 (0.6–4.8)	0.28
PLNM	2.6 (1.2–5.7)	0.01	2.7 (1.1–5.2)	0.03	1.7 (0.9–3.3)	0.10	1.5 (0.8–2.9)	0.21

*ENE, extranodal extension; HR, hazard ratio; ILNM, ingunal lymph node metastasis; MFS, Metastasis-free survival; OS, Overall survival; PLNM, pelvic lymph node metastasis*.

### Metastasis-Free Survival

**Figure 4:**
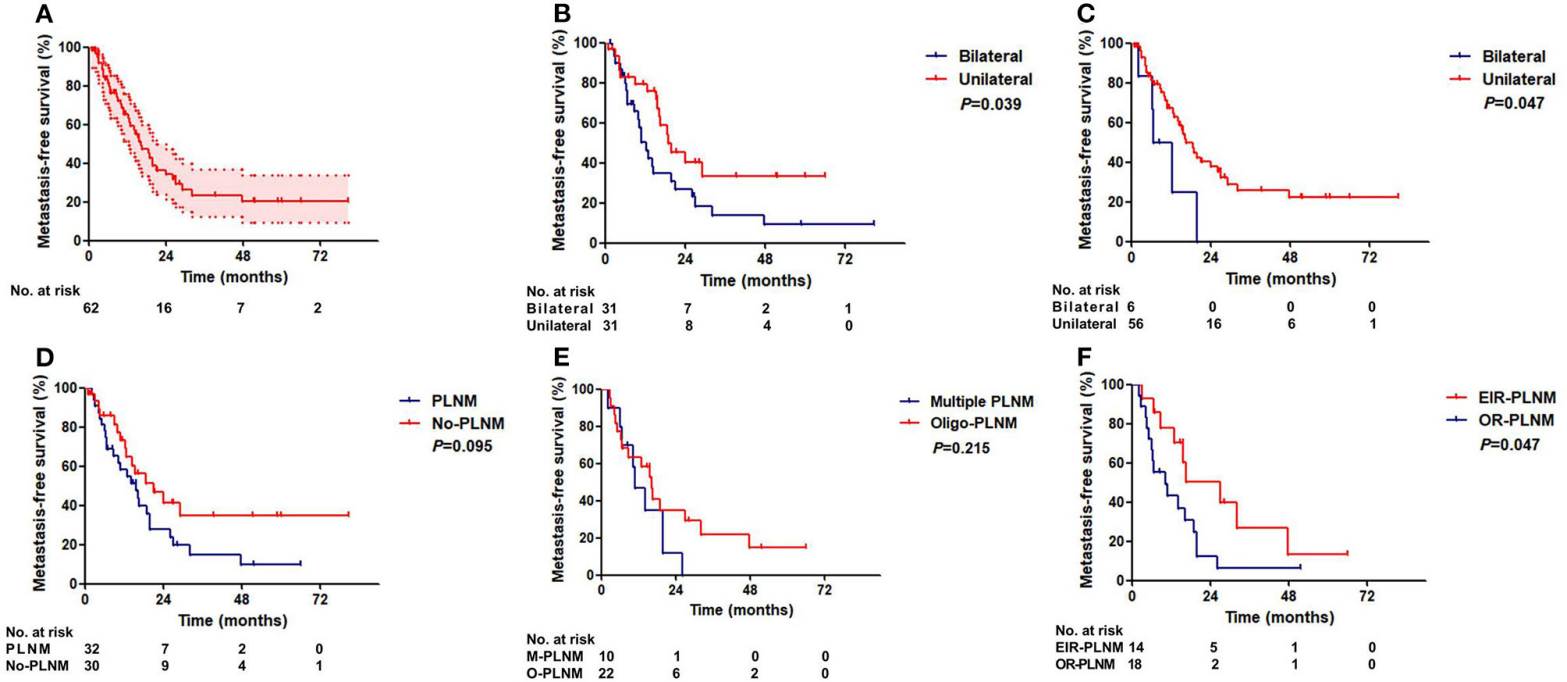
Metastasis-free survival of ENE patients. **(A)** All patients. **(B)** Patients with unilateral and bilateral inguinal LNM. **(C)** Patients with unilateral and bilateral ILN-ENE. **(D)** Patients with and without PLNM. **(E)** Patients with multiple PLNM and oligo-PLNM. **(F)** Patients with external iliac region-PLNM and other region-PLNM (EIR-PLNM, external iliac region-PLNM; OR-PLNM, other region PLNM).

The median metastasis-free survival (MFS) was 16.1 months (*95% CI:11.4–20.8*). The 3-year MFS rate was 23.4% (*95% CI: 11.7–36.1%*, Figure 4A).

Patients with bilateral ILNM exhibited worse MFS rates than patients with unilateral ILNM (36-month MFS: 33.5 vs. 13.9%, *P* = *0.039*, Figure 4B). Patients with unilateral ILN-ENE exhibited better MFS than patients with bilateral ILN-ENE (36-month MFS: 25.8% vs. 0, *P* = *0.047*, Figure 4C).

No significant differences in survival were found between PNLM patients with or without multiple PLNM (Figures 4D,E). Patients with external iliac region-PLNM exhibited significantly better MFS than patients with PLNM in other regions (36-month MFS: 26.7 vs. 6.2%, *P* = *0.047*, Figure 4F). Multivariate Cox regression analysis results are shown in Table 4.

## Discussion

Lymph node status is a strong prognostic factor in patients with PSCC (1, 2). PLND performed in penile cancer patients can improve patient survival and facilitate more precise staging (5, 6). However, little information about the necessary anatomic extent of PLND and reliable staging is available (1–3, 14, 15). We investigated the primary lymphatic landing sites and outcomes in patients with ILN-ENE from multiple centers to determine the necessary extent of PLND in patients with PSCC.

Two major controversies exist regarding penile PLND. One is whether PLND should be performed ipsilaterally or bilaterally in patients with unilateral ILNM. In one small cohort study, bilateral PLND was associated with improved survival in patients with unilateral ILNM (5). However, 38 (75%) patients underwent ipsilateral PLND, and only 13 (25%) underwent bilateral PLND, potentially leading to coincidental findings in this study. The hypothesis generated by these results must be further confirmed in larger series. However, the stepwise disseminative pattern of metastasis in penile cancer was confirmed (4, 6). There is no direct lymphatic drainage from penile tumors to pelvic LNs, and lymphadenectomy is therefore not indicated if there is no involvement of the inguinal nodes on that side (16). A majority of researchers' recommendations for ipsilateral PLND have been based on retrospective studies (1, 17). This recommendation is supported by our study.

Our multicenter data also demonstrated that no PLNM was identified in the absence of inguinal LN involvement. The external iliac and obturator region were the most commonly affected regions for ENE patients, and the only affected regions in patients with oligo-PLNM. However, patients with external iliac region PLNM had better OS and MFS. These results suggest that patients with external iliac region PLNM, especially oligo-PLNM, had anticipated outcomes with PLND. Further analysis of the mechanisms of metastasis in PSCC may help clarify the results of this study.

Another controversy involves the necessary extent of PLND (1, 2, 18). The dissection extent is controversial, and no dissection template has been universally accepted. According to Campbell–Walsh Urology, pelvic lymphadenectomy should include the distal common iliac, external iliac, and obturator nodes (3). According to the NCCN guidelines, PLND should extend to the external iliac, internal iliac, and obturator nodes (2). Penile cancer with involvement of pelvic lymph nodes is relatively uncommon; therefore, urologists at different centers have no common direct evidence to assess the value of one dissection template over another (19). Currently, studies have identified only pathological parameters of inguinal lymph node involvement to predict pelvic LN involvement (20–22).

To the best of our knowledge, the current study is the first to assess anatomic aspects of patients with ENE treated with PLND. Previous studies have shown that ENE was associated with PLNM (2, 14, 15). In a previous anatomical study, Zhu et al. noted that the external iliac region was the most commonly involved, and the obturator region was less commonly involved (23). However, the number of patients in this study was relatively small (seven patients with PLNM), and only three nodal regions were considered for iliac lymphadenectomy: the external iliac, obturator, and common iliac regions.

In our analysis, 62 eligible patients underwent PLND and were included in the analysis of the distribution. Our findings also showed that patients with bilateral ILNM had a high risk of PLNM (especially multiple metastases) and had worse survival. Additionally, patients with unilateral ILN-ENE also exhibited significantly better OS than patients with bilateral ILN-ENE. Therefore, we presumed that heterogeneity exists in ILNM patient staging. The inguinal LNM stage may have a positive predictive effect on survival for micrometastasis or tumor load (4, 6). Patients with bilateral inguinal LNM needed more extensive surgery according to the PLND, potentially reducing the tumor burden. For example, the “ENE patients with bilateral ILNM” template should be extended to include the external iliac, obturator, common iliac, and internal iliac regions in ILN-ENE patients.

Our study has the following limitations. (1) The data collection was retrospective, and the study period was short. (2) The number of cases was relatively small. Oncology results should be taken with caution because of short follow-up time. (3) We did not report perioperative complications. Surgical complications might impact survival. We firmly believe that the persuasive power of the results will greatly increase with further research. (4) Other variables that may influence the prognosis were not examined. For example, patients with ENE should be treated with neoadjuvant/adjuvant chemotherapy or radiation therapy, which might affect the prognosis. Due to the limited number of included studies, various chemotherapy regimens, and lack of article information, we did not perform subgroup analysis. We believe that this type of analysis will be important in future validation studies. (5) A larger proportion of patients who underwent bilateral PLND were included, which may have resulted in overtreatment. However, the EAU and NCCN guidelines are controversial with regard to bilateral PLND. (6) The skill of surgeons who performed PLND may not have been uniform in all centers. We declare that all analyses were considered exploratory rather than hypothesis-based.

## Conclusion

In summary, the external iliac and obturator region were the most commonly affected regions in ENE patients, and these regions were the only affected regions in patients with oligo-PLNM. Patients with bilateral ILNM had a high risk of PLNM and worse survival. These findings suggest that patients with external iliac region PLNM, especially oligo-PLNM, had better outcomes with PLND, and patients with bilateral ILNM need more extensive PLND, potentially reducing the tumor burden.

## Data Availability Statement

The raw data supporting the conclusions of this article will be made available by the authors, without undue reservation.

## Ethics Statement

Written informed consent was obtained from the individual(s) for the publication of any potentially identifiable images or data included in this article.

## Author Contributions

Z-SL, HH, Y-HL, and F-JZ designed the study. Z-SL and Y-HL analyzed and interpretated the data and wrote the manuscript. Z-SL, Y-HL, F-JZ, and HH provided valuable insights into data interpretation and manuscript writing. All authors collected the data, contributed to the article and approved the submitted version.

## Conflict of Interest

The authors declare that the research was conducted in the absence of any commercial or financial relationships that could be construed as a potential conflict of interest.

## Abbreviations

EAU: European Association of Urology
ENE: extranodal extension
ILN: inguinal lymph node
ILND: inguinal lymph node dissection
LN: lymph node
LNM: lymph node metastasis
NCCN: National Comprehensive Cancer Network
PLND: pelvic lymphadenectomies
PLNM: pelvic lymph node metastasis
PSCC: penile squamous cell carcinoma.
